# Improved late gadolinium enhancement imaging of left ventricle with isotropic spatial resolution

**DOI:** 10.1186/1532-429X-14-S1-O22

**Published:** 2012-02-01

**Authors:** Mehmet Akcakaya, Hussein Rayatzadeh, Susie Hong, Thomas H Hauser, Raymond H Chan, Tamer A Basha, Kraig V Kissinger, Beth Goddu, Warren J Manning, Reza Nezafat

**Affiliations:** 1 Medicine, Beth Israel Deaconess Medical Center, Harvard Medical School, Boston, MA, USA; 2 Radiology, Beth Israel Deaconess Medical Center, Harvard Medical School, Boston, MA, USA

## Background

Recent studies have shown the prognostic value of the infarct border zone of late gadolinium enhancement (LGE) images in patients with myocardial infarction [1]. This border zone has also been associated with ventricular arrhythmia [2,3]. The accuracy of the characterization of this area depends on spatial resolution of the imaging. 3D LGE allows improved spatial resolution, especially in through-plane direction. However imaging with an isotropic spatial resolution necessitates very long scan time. In this study, we sought to investigate if compressed-sensing (CS) based image acceleration method [4] allows LGE imaging with isotropic spatial resolution.

## Methods

A prospective random under-sampling LGE acquisition was implemented on 1.5T Philips scanner. A free-breathing ECG-triggered inversion-recovery GRE sequence with navigator-gating was used for all acquisitions on 18 patients (5 females, 52.8±16.3 years) 10 to 20 minutes after bolus infusion of contrast agent. Each subject were imaged using two LGE sequence in random order: a) a 3-fold-accelerated LGE scan with isotropic spatial resolution of 1.2-to-1.7 mm^3^, b) LGE scan with non-isotropic resolution of 1.7×1.7×4.0mm^3^ were performed with imaging parameters of TR/TE/α=5.2/2.6ms/25°, FOV=320×320×100mm^3^. Random undersampling was implemented as described in [5], where the central k-space (45×35 in ky-kz) was fully-sampled. Acquisition times were 3 mins assuming 100% scan efficiency at 70 bpm for both scans. The images from the accelerated scans were reconstructed using an advanced CS-technique, called LOST [4].

## Results

**Figure 1:**
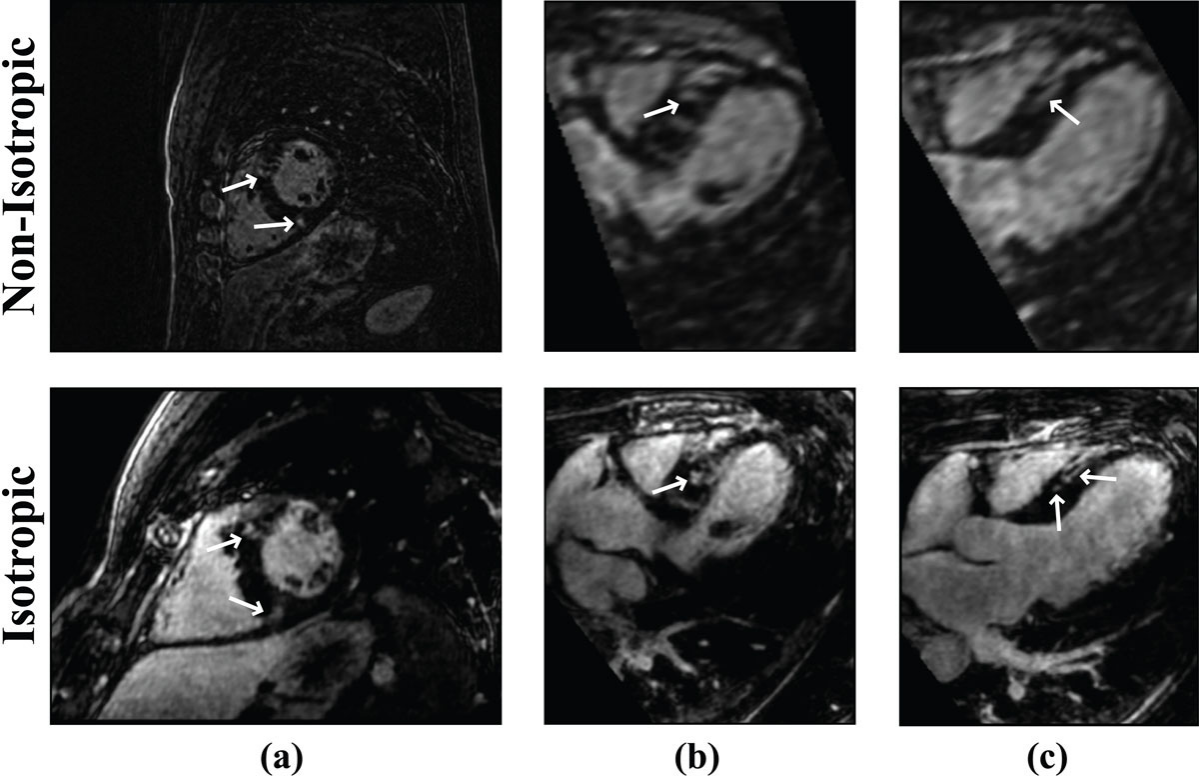
Reformatted LGE images from a patient with HCM, acquired using non-isotropic spatial resolution (top), and isotropic spatial resolution (bottom). An isotropic resolution allows better visualization of scar morphology in images acquired using LOST-accelerated acquisition.

**Figure 2:**
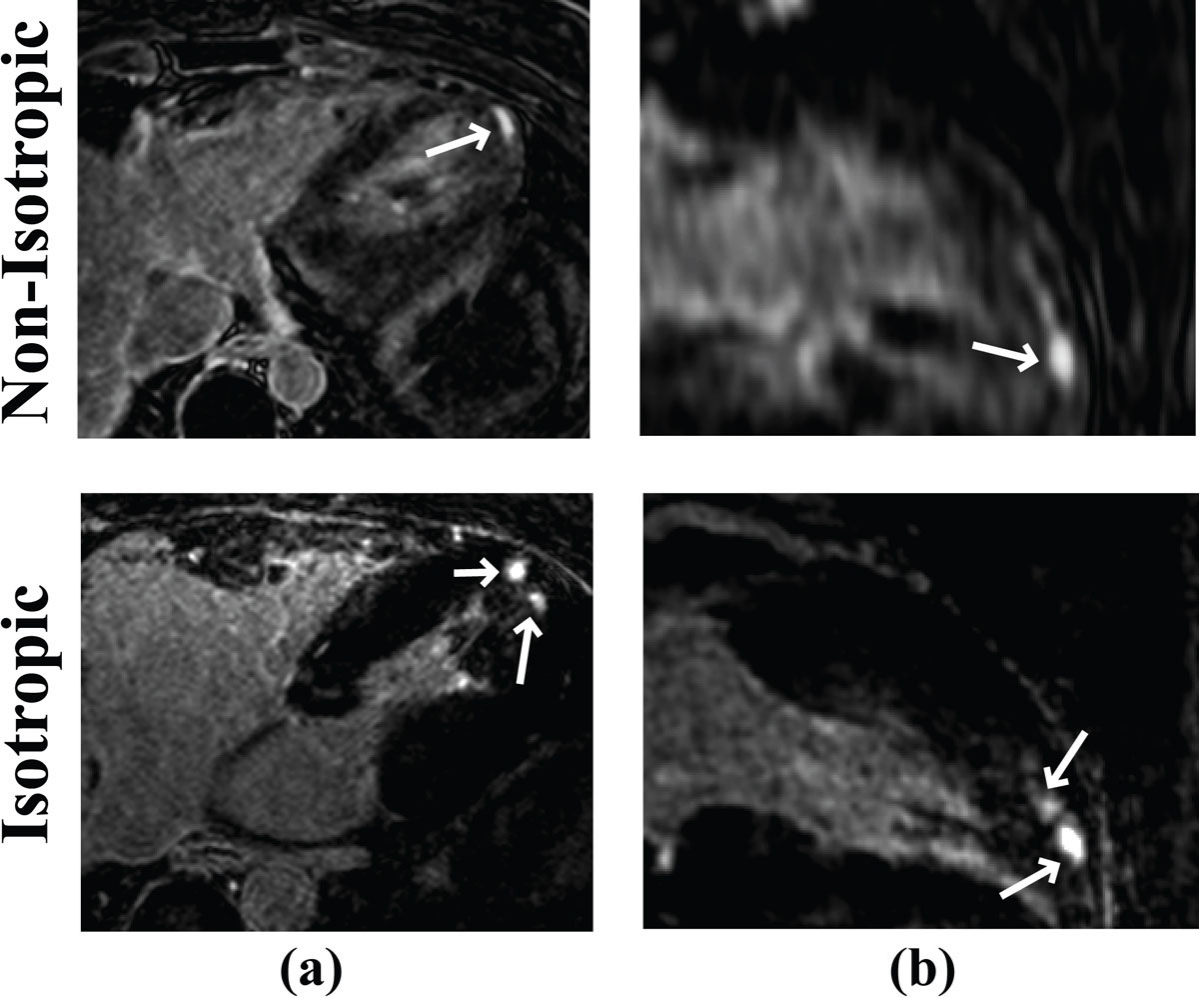
Axial (left) and reformatted long-axis (right) LGE images from a patient. Non-isotropic LGE was acquired with a spatial resolution 1.7×1.7×4.0 mm^3^ (top), whereas LOST-reconstructed isotropic resolution images from the accelerated scan had a resolution of 1.2×1.2×1.2 mm^3^ (bottom).

Figure 1 shows LGE images from a patient with hypertrophic cardiomyopathy acquired using two different approaches. An improved isotropic spatial resolution allows better characterization of the scar morphology. Figure 2 shows another example in a patient undergoing ICD implantation as a primary prevention of sudden cardiac death.

## Conclusions

Accelerated LGE imaging with isotropic spatial resolution allows improved visualization of scar morphology. Further quantitative measurements of infarct border zones in a larger cohort of patients are needed to better understand the prognostic value of the improved scar imaging.

## Funding

NIH R01EB008743-01A2.
